# Analysis of differentially expressed genes responsible for the suppressive effect of anisomycin on cell proliferation of DLD-1 cells

**DOI:** 10.1016/j.bbrep.2021.101038

**Published:** 2021-06-05

**Authors:** Hironori Ushijima, Rina Monzaki, Mika Funakoshi

**Affiliations:** Department of Analytical Biochemistry, School of Pharmacy, Iwate Medical University, 1-1-1, Idaidori, Yahaba, Shiwa-gun, Iwate, 0283694, Japan

**Keywords:** Anisomycin, DLD-1, RNA-seq, LAMB3, ATF3, DLD-1, a colorectal adenocarcinoma cell line isolated by D. L. Dexter and associates, DMSO, dimethyl sulfoxide, FBS, fetal bovine serum, JNK, c-Jun N-terminal kinase, PBS, phosphate buffered saline, RNA-seq, RNA sequencing, CPM, counts per million, GO, gene ontology, KEGG, Kyoto Encyclopedia of Genes and Genomes

## Abstract

Anisomycin is used as a chemical compound that possesses c-Jun N-terminal kinase (JNK)-activating effects. Recently, the potent anti-tumor effects of anisomycin have received much attention. In addition to its JNK-activating effects, anisomycin has been reported to affect gene expression in osteosarcoma, leukemia, hepatocellular carcinoma, ovarian cancer and other cancers. We previously demonstrated that anisomycin induced the degradation of transcription factor GATA-6 in DLD-1 cells (a colorectal cancer cell line) and inhibited their proliferation. However, the details of the gene network involved in the process remain unclear. In this study, we conducted an RNA-seq analysis of differentially expressed genes (DEGs) in anisomycin-treated DLD-1 cells to identify the molecular process of growth-suppressive genes. We found that LAMB3, which regulates cell adhesion and migration, and NFKB2 were down-regulated by anisomycin. In addition, the mRNA expression of several tumor suppressor genes (ATF3, ERRFI1, KLF6, and AKAP12) was transiently enhanced at 3 h after anisomycin treatment. These results suggest that anisomycin blocks a PI3K/Akt-signaling cascade to lead to the suppression of cell growth.

## Highlights

• There were 396 down-regulated genes and 504 up-regulated genes (fold change value > 2) in anisomycin-treated cells.
• LAMB3 and LAMA3, associated with cell adhesion and migration, were down-regulated at 24 h after anisomycin treatment.
• The 4 tumor suppressor genes (ATF3, ERRFI1, KLF6, AKAP12) were transiently up-regulated at 3 h after anisomycin treatment.

## 1 Introduction

Anisomycin, which was originally identified as an antibiotic compound extracted from *Streptomyces griseolus* [1,2], is widely used in biological experiment both *in vitro* and *in vivo* due to its wide variety of bioactivities. It was previously reported that anisomycin negatively regulated eukaryotic protein synthesis via inhibition of peptidyl transferase in ribosomes [3]. Anisomycin has also been shown to activate both mitogen activated protein kinase (MAPK) and c-Jun N-terminal kinase (JNK) [4]. Recently, the potent anti-tumor effects of anisomycin have attracted a great deal of interest. It was reported that anisomycin treatment led to dysfunction of mitochondria and inhibited their biogenesis in osteosarcoma cells [5]. In chronic phase chronic myeloid leukemia cell lines, anisomycin inhibited cell growth and, when used in combination with imatinib, achieved a greater anti-tumor effect than either agent alone [6]. Anisomycin treatment in hepatocellular carcinoma cells resulted in an increase in the expression levels of CD58 recognized by natural killer cells [7]. It was also reported that the induction of long non-coding RNA BACE1-AS by anisomycin stabilized the expression of BACE1 through a feed-forward mechanism and contributed to the suppression of cell proliferation in ovarian cancer stem cells [8].

We have reported that anisomycin potently inhibited the proliferation of colorectal cancer-derived DLD-1 cells under planar culture or spheroid culture conditions through induction of proteolysis of transcription factor GATA-6 [9]. Furthermore, an enhanced anti-tumor effect of 5-fluorouracil was observed by co-treatment with anisomycin. GATA-6 may be an effective target of colorectal cancer cells [10,11], but in general, the region downstream of transcription factors such as GATA-6 encodes a vast protein network. Our previous experimental data [9] could draw only a small part of the overall picture of the molecular mechanisms underlying the proteasome-associated degradation of GATA-6.

Therefore, in this study, we conducted an RNA-seq analysis of differentially expressed genes (DEGs) in anisomycin-treated DLD-1 cells in order to elucidate the molecular process of growth-suppressive genes. We identified 396 down-regulated genes and 504 up-regulated genes in anisomycin-treated cells, and found that these genes were involved in variety of intracellular biological processes. Among the genes down-regulated by anisomycin, LAMB3, which encodes a protein belonging a cluster that functions in cell adhesion and migration, showed the greatest decrease in expression. Our findings suggest that tumor suppressive genes, such as ATF3, were activated early in the sequence of events leading to the inhibitory effect of anisomycin on cell proliferation.

## 2 Materials and methods

### 2.1 Materials

RPMI1640, trypsin and FBS were purchased from Thermo Fisher Scientific Inc. DMSO and anisomycin were supplied by Nacalai Tesque Inc. (Kyoto, Japan) and FUJIFILM Wako (Osaka, Japan), respectively. All other chemicals used were of the highest grade commercially available.

### 2.2 Cell culture

DLD-1 cells were cultured in RPMI1640 medium containing 10% (v/v) FBS and antibiotics (0.07 g/L penicillin G, and 0.1 g/L streptomycin) at 37 °C. The proliferation of DLD-1 cells in the presence of anisomycin was monitored by cell counting with a TC10 automated cell counter (BIO-RAD). Briefly, DLD-1 cells were seeded onto 100 mm dishes (1.5 × 10^6^ cells/dish). After 24 h, the medium was changed to fresh medium containing 0.5 μM anisomycin, followed by further culture for the indicated times. Cells were collected by 0.25% trypsin treatment at the indicated time points and then washed with PBS. An aliquot of a cell suspension (10 μL) was transferred to a cell counting slide for the cell counting by TC10.

### 2.3 RNA sequencing

RNA sequencing was performed using a Novaseq 6000 (Illumina) sequencer according to the manual of Rhelixa Co., Ltd (Tokyo, Japan). The total RNA molecules were extracted from DLD-1 cells with or without 0.5 μM of anisomycin using an RNeasy extraction kit (QIAGEN). The total RNA samples, that showed an RNA integrity number (RIN) > 7 determined by a Bioanalyzer (Agilent), were subjected to cDNA library preparation. Then 2 μg of total RNAs were converted to cDNAs with oligo dT primers using a NEBNext Poly (A) Magnetic Isolation Module (for poly A selection, NEB) and a NEB Next Directional Ultra RNA Library Prep Kit for Illumina (for strand specific libraries, NEB). These cDNA libraries were sequenced with a NovaSeq 6000 system (Illumina) to produce 150 base pair-end reads.

### 2.4 Identification of DEGs and pathway analysis with bioinformatic databases

After quality control of sequenced reads with FastQC (ver. 0.11.7) [12], extra bases, such as adapter sequences in the filtered reads, were trimmed with Trimmomatic (ver. 0.36) [13]. The Genome mapping of sequenced reads was performed with HISAT2 (ver. 2.1.0) [14]. The read count files containing values of mapped reads were loaded in iDEP platform (ver. 0.91) [15], then the read count values were normalized using the Relative Log Expression (RLE) method implemented in the DESeq2 [16]. The analysis of variance of read counts data in multiple cell groups (different cultivation time in the presence of anisomycin) was performed according to the previous procedure [15].

To identify DEGs (between control group and anisomycin-treated group, 3 samples in each group), the cutoff value of false discovery rate and the minimum fold change value were set to 0.001 and 2, respectively as threshold values. Then the DESeq2 program on iDEP was executed. The identified DEGs were entered into a pathway analysis conducted using two bioinformatic databases (GO biological process database [17] and KEGG pathway database containing the network for metabolism, genetic information processing, environmental information processing, cellular processes, organismal systems and human disease [18]), then the adjusted *P* values in each category were calculated.

### 2.5 Statistical analysis

Statistical analysis was conducted with R programing scripts. The Student *t*-test was performed to determine significant differences between two groups in the growth curve of DLD-1 cells (control group vs anisomycin-treated group, 3 samples in each group) and in the comparative analysis of normalized counts of DEGs (control group vs anisomycin-treated group, 3 samples in each group). The one-way analysis of variance with Bonferroni correction was done in the comparative analysis of normalized counts of tumor suppressor genes among multiple groups; 24 h-group which was incubated with anisomycin for 24 h vs 3 h- (or 6 h-, 12 h-) groups which were incubated with anisomycin for 3 h (or 6 h, 12 h). The 3 samples were prepared in each group. The *P* < 0.05 was considered to be statistically significant.

## 3 Results

### 3.1 Enrichment analysis of DEGs by RNA-seq in DLD-1 cells treated with anisomycin

**Fig. 1:**
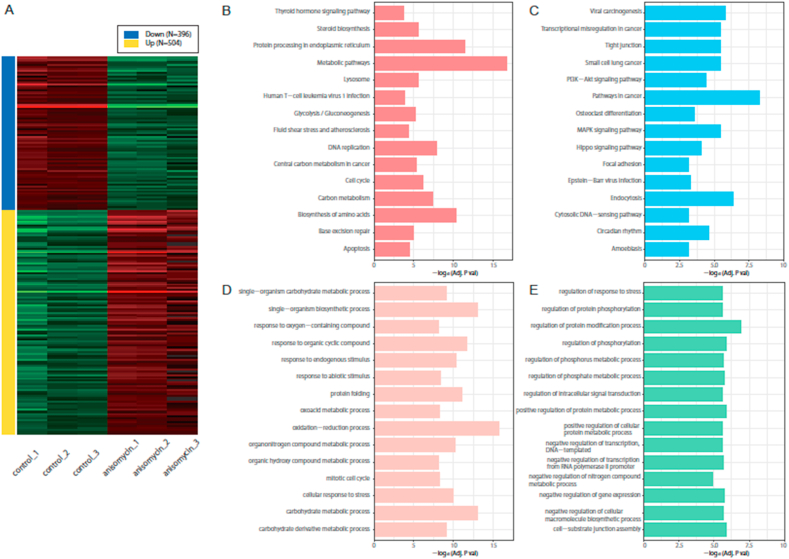
Enrichment and pathway analysis of DEGs in DLD-1 cells treated with anisomycin. (A) Heatmap showing the hierarchical clustering of differentially expressed genes derived from DLD-1 cells, which were treated with or without 0.5 μM anisomycin for 24 h. (B) The up-regulated genes and the down-regulated genes (shown in C) were subjected to pathway analysis with KEGG databases. The bar length represents the strength of the relationship in each category. (D) The biological processes of up-regulated genes and down-regulated genes (E) were analyzed using GO databases.

**Supplemental Fig. 1:**
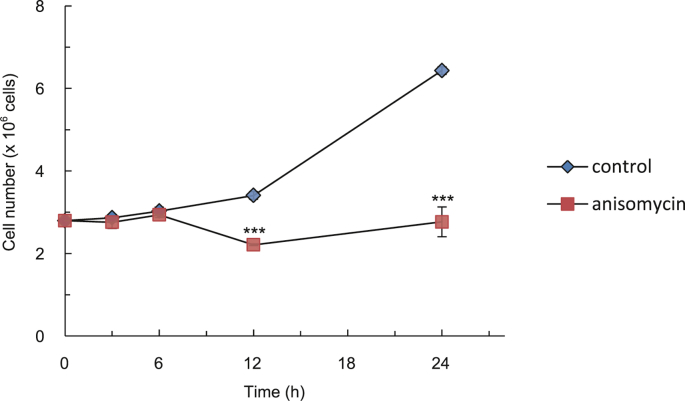
The proliferation of DLD-1 cells was suppressed by anisomycin. DLD-1 cells were cultured in the medium with or without 0.5 μM anisomycin for the indicated amounts of time. Cells were collected by trypsin treatment and the number of living cells was counted with an automated cell counter. Values are mean ± SD (*n* = 3). Statistical analysis was performed using Student *t*-test and significance was set as follows: ***P < 0.005.

DLD-1 cells were cultured in medium with or without 0.5 μM of anisomycin for 24 h (Supplemental Figs. 1) and a growth inhibitory effect of anisomycin was observed as previously reported [9]. We then extracted the total RNAs from the DLD-1 cells to perform RNA-seq analysis. In the enrichment analysis to compare paired DEGs (the DEGs in control cells and its counterpart in anisomycin-treated cells), we set fold change value at a threshold of more than 2. The heatmap data indicated that there were 396 down-regulated genes and 504 up-regulated genes in anisomycin-treated cells compared with non-treated control cells (Fig. 1A). These selected genes were subjected to pathway analysis with the KEGG and GO databases. The KEGG and GO pathways which satisfied the adjusted *P* value is less than 0.05 were considered to be significantly enriched. From the analysis with the KEGG database, it revealed that the intracellular biological pathways, in which up-regulated genes were involved, included the metabolic pathways, protein processing in endoplasmic reticulum (ER), biosynthesis of amino acids, DNA replication, and carbon metabolism (Fig. 1B). It also showed that the pathways related to cancer, endocytosis, viral carcinogenesis, transcriptional misregulation, tight junction, focal adhesion, small cell lung cancer and circadian rhythm were included in the intracellular biological pathways involved in the down-regulated genes treated by anisomycin (Fig. 1C). Some cell proliferation-related signaling pathways, such as MAPK, PI3K-Akt, and Hippo signaling, were found to be included in the pathways involving the down-regulated genes. Using GO biological process databases, it resulted that the biological processes in the up-regulated genes included oxidation-reduction process, single-organism biosynthetic process, response to organic cyclic compound process, protein folding process, response to endogenous stimulus process, mitotic cell cycle process and cellular response to stress process (Fig. 1D). The metabolic processes, one of the pathways shown in the analysis using KEGG databases, were also shown in the analysis using GO biological process databases. The metabolic processes related to carbohydrate, organonitrogen compound, organic hydroxyl compound and oxoacid were included in the processes involving the up-regulated genes. We also found that the biological pathways involving the down-regulated genes group included the regulation processes related to stress response, protein phosphorylation, protein modification, phosphate metabolism, protein metabolism, signal transduction and gene expression (Fig. 1E).

### 3.2 Changes in the gene expression of anisomycin-treated cells over 24 h

**Fig. 2:**
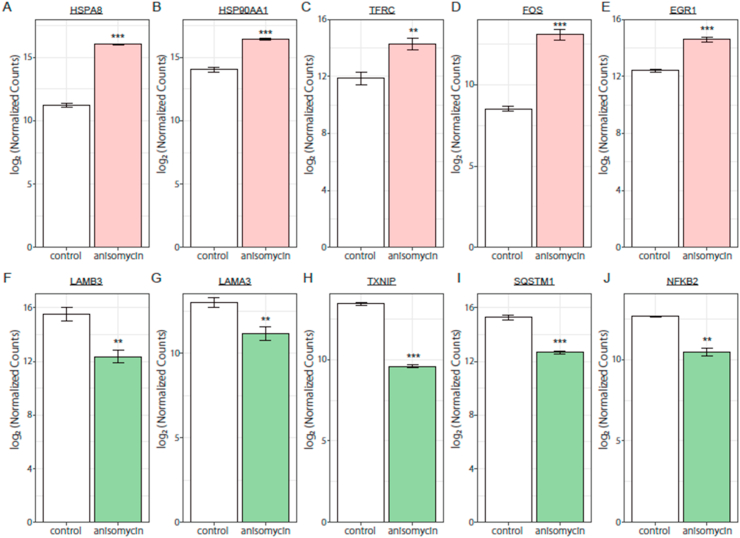
Up- or down-regulated genes in DLD-1 cells treated with anisomycin for 24 h. (A-E) The upper panel shows particular genes that showed high variation of fold change values and formed a protein network cluster in the up-regulated genes group. The same analysis was performed in down-regulated genes, and the results are shown in the lower panel (F-J). Values indicate the mean of log_2_ of the normalized counts ± SD (*n* = 3). Statistical analysis was performed using Student *t*-test and significance was set as follows: **P < 0.01, ***P < 0.005.

**Supplemental Fig. 2:**
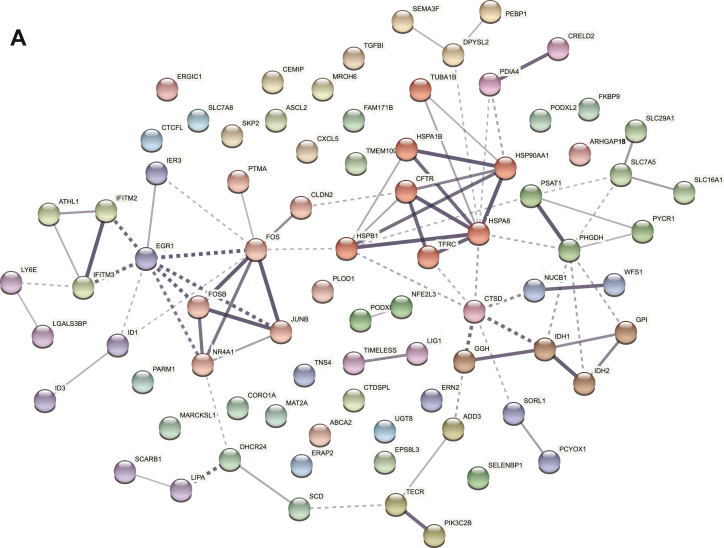
Anisomycin-induced protein-protein interactions from the STRING database. (A) The top 80 genes with high fold change values in the up-regulated genes group are displayed in figure. (B) The low fold change group also showed 80 genes. Each node is linked if there are functional or physiological associations between proteins. The line thickness indicates the strength of data support and the dotted line indicates the interactions between network clusters.

In order to elucidate the genes responsible for the potent growth-suppressive effect of anisomycin, we analyzed the up or down regulated genes from the viewpoint of protein-protein interaction using STRING databases. The top 80 genes, arranged in descending order of the fold change value in the up-regulated genes, were represented as node shapes in the network (Supplemental Fig. 2A). The 80 down-regulated genes were also represented with the same shape (Supplemental Fig. 2B). We noted the particular genes that showed a high variation of fold change values (listed in Table 1) and formed a protein network cluster. HSPA8, belonging to the HSP70 family, was the most up-regulated of the genes affected by anisomycin (the fold change value compared with the control was approximately 28, calculated by normalized counts) (Fig. 2A). The other up-regulated genes were HSP90AA1, TFRC (transferrin receptor 1), FOS, and EGR1 (early growth response 1), respectively (Fig. 2B–E). LAMB3 (laminin subunit beta-3), which is associated with cell migration and cell attachment processes, was the most down-regulated gene (the fold change value compared with the control was approximately 0.11). The genes, LAMA3 (laminin subunit alpha 3), TXNIP (thioredoxin interacting protein), SQSTM1 (sequestosome 1), and NFKB2 (nuclear factor kappa B) were also down-regulated and formed a protein-protein interaction cluster (Fig. 2F–J).

**Table 1:** The fold change values of up- or down-regulated genes in anisomycin-treated DLD-1 cells compared with control cells.

	Genes	Fold change
Up-regulated genes	HSPA8	28.4
	HSP90AA1	5.4
	TFRC	5.4
	FOS	23.3
	EGR1	4.5
Down-regulated genes	LAMB3	0.11
	LAMA3	0.28
	TXNIP	0.070
	SQSTM1	0.17
	NFKB2	0.22

### 3.3 Induction of tumor suppressor genes by anisomycin treatment

At 24 h after incubation with anisomycin, it appeared that the DEGs among the up-regulated genes were associated with promotion rather than suppression of cell proliferation. In order to reveal the presence of tumor suppressor genes induced by anisomycin, we additively performed the identification of DEGs using anisomycin-treated cells with different incubation times. The normalized counts of tumor suppressor genes in the cells treated with anisomycin for less than 12 h were compared with those in the cells treated with anisomycin for 24 h (Fig. 3). The results showed that there were four tumor suppressor genes that exhibited higher expression at 3 h than at 24 h of anisomycin treatment and we observed the tendency for the expression levels of them to be decreased in a time-dependent manner (from 3 h to 24 h). These four genes appeared to be transiently induced, such that their expression levels were decreased at 24 h. ATF3 (activating transcription factor 3) was the gene with the largest variation in fold change value (approximately 41) when comparing the 3 h treatment group with the 24 h treatment group. The highest fold change values of other 2 genes were also observed at the same comparing time (3 h vs 24 h) as follows: ERRFI1 (ERBB receptor feedback inhibitor 1), 27; KLF6 (Kruppel-like factor 6), 20. The expression pattern of AKAP12 (A kinase-anchoring protein) showed different tendency comparing with other 3 genes and its highest fold change value was 22 at 6 h after treatment of anisomycin. The KEGG pathway analysis for the anisomycin-treated groups with different incubation time suggested that some pathways associated with tumor suppressive genes, such as TNF signaling, apoptosis, NF-kappa B pathways, and Hippo signaling pathways might be activated by anisomycin-treatment at 3 h and 6 h. The metabolic pathways and ER associated-protein processing might be negatively affected at 12 h after incubation of anisomycin (Supplemental Table 1).

**Fig. 3:**
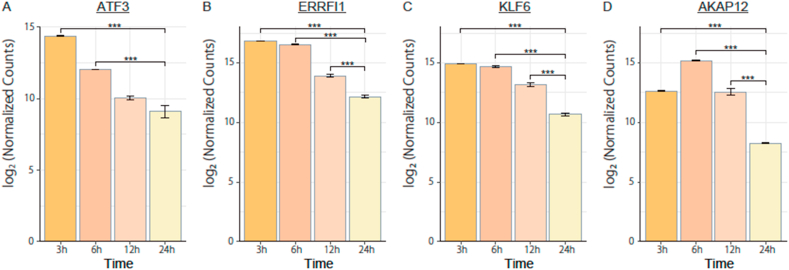
Expression of tumor suppressor genes by anisomycin treatment. DLD-1 cells were treated with 0.5 μM anisomycin for the indicated amounts of time, and then their total RNAs were collected for RNA-seq analysis. (A-D) The up-regulated expression of tumor suppressor genes was compared between two anisomycin-treated groups with different incubation times. Values indicate the mean of log_2_ of the normalized counts ± SD (*n* = 3). Statistical analysis was performed using one-way analysis of variance with Bonferroni correction and significance was set as follows: ***P < 0.005.

## 4 Discussion

Anisomycin has been proven useful as a JNK (c-Jun N-terminal kinase) activator to elucidate gene networks mainly in the field of MAPK-associated signal transduction research [4]. Recently, anisomycin has been attracting attention for its potent anti-tumor effects toward a variety of cancer cells, such as osteosarcoma cells, chronic phase chronic myeloid leukemia cell lines, hepatocellular carcinoma cells, and ovarian cancer stem cells [[5], [6], [7], [8]]. Anisomycin is thus a very promising therapeutic agent for use in cancer chemotherapy.

In this study, we performed RNA-seq analysis in order to comprehensively analyze the DEGs, in anisomycin treated DLD-1 cells, and thereby elucidate the genes play an important role in suppressing cell proliferation. At 24 h after treatment with anisomycin, the genes that were up-regulated compared with control cells shared the characteristic of promoting cell growth. HSPA8 and HSP90AA1, representative molecular chaperones, have a wide variety of effects, such as prevention of unfolded-protein aggregation, chaperone-mediated autophagy, and promotion of tumor cell proliferation [19]. TFRC has been reported to promote tumor migration via the positive regulation of AXIN2 [20]. FOS, also known as AP-1 subunit, is a major proto-oncogene [21], EGR1 has been reported to activate the transcription of miR-20b and thereby inhibit the expression of PTEN (a tumor suppressor gene) in breast cancer [22]. These genes and TXNIP [23] may serve coordinately to mount a compensatory response against the cellular stresses caused by the growth-suppressive effect of anisomycin. On the other hand, the down-regulated genes (other than TXNIP) may also play a role in mediating the growth-suppressive effect of anisomycin. It has been reported that LAMB3 overexpression is a marker of poor prognosis in colorectal cancer, and that LAMB3 promotes migration *in vitro* and tumor growth and metastasis *in vivo* [24]. Down regulation of LAMB3 and LAMA3, which are components of Laminin-332 protein [25], may contribute to the effects of anisomycin. Moreover a decrease of SQSTM1, (also known as p62), which plays a key role in autophagy, might inhibit the NFKB2 signaling pathway, which could result in potent arrest of the cell progression [26].

We considered the possibility that some genes negatively controlled the expression of LAMB3 and other genes earlier than 24 h after cultivation with anisomycin, and we identified 4 tumor suppressor genes at short cultivation time with anisomycin (at 3 h). Although the expression of AKAP12 showed interesting pattern that the expression seemed to be increased at 12 h before decrease, we think further experiments considering time-series linked to the downstream of AKAP12 signaling are needed to explain its phenomenon. Regarding the role of KLLF6, it was possible that up-regulated KLF6 depressed the cell migration and invasion activity through the inhibition of matrix metalloproteinase 9 [27]. Anisomycin was previously shown to induce ATF3 expression [28], and it was also reported that ATF3, which is known to serve as a stress sensor protein, suppresses IL-6 expression via the negative regulation of NFKB2 [29]. In regard to signal transduction systems, it has been suggested that the PI3K/Akt pathway is one of the major pathways that play a central role as a potent growth accelerator for various cancer cells. Recent reports suggest that ATF3, ERRFI1, and AKAP12 negatively control the activity of Akt [[30], [31], [32]], which may suggest that anisomycin inhibited the Akt-associated cascades at 3 h after treatment.

In summary, we analyzed DEGs in anisomycin treated DLD-1 cells for the first time and found that several tumor-suppressor genes, including ATF3, were transiently induced at 3 h after treatment. This suggested that the reduced expression levels of LAMB3 and LAMA3 led to low adhesion and migration activity, and that these changes, together with a simultaneous decrease of NFKB2, were mainly responsible for the growth-suppressive effect of anisomycin.

## Credit authorship contribution statement

**Hironori Ushijima:** Conceptualization, Methodology, Investigation, Resources, Data curation, Validation, Formal Analysis, Visualization, Supervision, Writing, Funding acquisition, Project administration. **Rina Monzaki:** Methodology, Investigation, Data curation, Validation, Formal Analysis, Visualization. **Mika Funakoshi:** Investigation, Data curation, Visualization.

## Declaration of competing interest

The authors declare that there are no conflicts of interest or any competing financial interest.

## Figures and Tables

**figs3:**
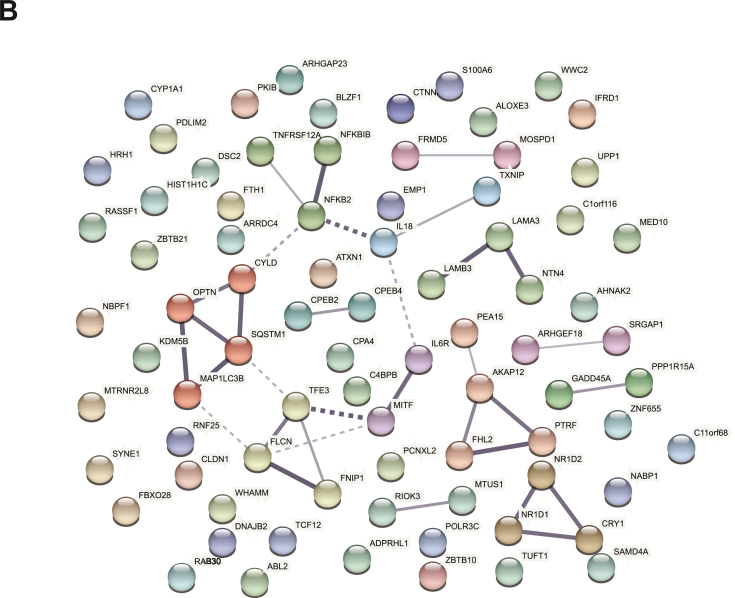


## References

[1] Sobin B.A., Tanner F.W. (1954). ANISOMYCIN, 1 a new anti-protozoan antibiotic. J. Am. Chem. Soc..

[2] Beereboom J.J., Butler K., Pennington F.C., Solomons I.A. (1965). Anisomycin. I. Determination of the structure and stereochemistry of anisomycin. J. Org. Chem..

[3] Grollman A.P. (1967). Inhibitors of protein biosynthesis. II. Mode of action of anisomycin. J. Biol. Chem..

[4] Liang S.-H., Zhang W., McGrath B.C., Zhang P., Cavener D.R. (2006). PERK (eIF2alpha kinase) is required to activate the stress-activated MAPKs and induce the expression of immediate-early genes upon disruption of ER calcium homoeostasis. Biochem. J..

[5] Cao C., Yu H., Wu F., Qi H., He J. (2017). Antibiotic anisomycin induces cell cycle arrest and apoptosis through inhibiting mitochondrial biogenesis in osteosarcoma. J. Bioenerg. Biomembr..

[6] Li Y., Hu J., Song H., Wu T. (2018). Antibiotic anisomycin selectively targets leukemia cell lines and patient samples through suppressing Wnt/β-catenin signaling. Biochem. Biophys. Res. Commun..

[7] Kim M., Lee S.-J., Shin S., Park K.-S., Park S.Y., Lee C.H. (2018). Novel natural killer cell-mediated cancer immunotherapeutic activity of anisomycin against hepatocellular carcinoma cells. Sci. Rep..

[8] Chen Q., Liu X., Xu L., Wang Y., Wang S., Li Q., Huang Y., Liu T. (2016). Long non-coding RNA BACE1-AS is a novel target for anisomycin-mediated suppression of ovarian cancer stem cell proliferation and invasion. Oncol. Rep..

[9] Ushijima H., Horyozaki A., Maeda M. (2016). Anisomycin-induced GATA-6 degradation accompanying a decrease of proliferation of colorectal cancer cell. Biochem. Biophys. Res. Commun..

[10] Zhao X., Zhang W., Ji W. (2018). miR-181a targets GATA6 to inhibit the progression of human laryngeal squamous cell carcinoma. Future Oncol..

[11] Tang J., Zhao J., Sheng W., Zhou J., Dong Q., Dong M. (2019). Ectopic expression of miR‐944 impairs colorectal cancer cell proliferation and invasion by targeting GATA binding protein 6. J. Cell Mol. Med..

[12] FastQC A quality control tool for high throughput sequence data. Babraham Bioinformatics Web site. http://www.bioinformatics.babraham.ac.uk/projects/fastqc/.

[13] Bolger A.M., Lohse M., Usadel B. (2014). Trimmomatic: a flexible trimmer for Illumina sequence data. Bioinformatics.

[14] Kim D., Langmead B., Salzberg S.L. (2015). HISAT: a fast spliced aligner with low memory requirements. Nat. Methods.

[15] Ge S.X., Son E.W., Yao R. (2018). iDEP: an integrated web application for differential expression and pathway analysis of RNA-Seq data. BMC Bioinf..

[16] Love M.I., Huber W., Anders S. (2014). Moderated estimation of fold change and dispersion for RNA-seq data with DESeq2. Genome Biol..

[17] Ashburner M., Ball C.A., Blake J.A., Botstein D., Butler H., Cherry J.M., Davis A.P., Dolinski K., Dwight S.S., Eppig J.T., Harris M.A., Hill D.P., Issel-Tarver L., Kasarskis A., Lewis S., Matese J.C., Richardson J.E., Ringwald M., Rubin G.M., Sherlock G. (2000). Gene ontology: tool for the unification of biology. The Gene Ontology Consortium. Nat. Genet..

[18] Kanehisa M., Goto S. (2000). KEGG: kyoto encyclopedia of genes and genomes. Nucleic Acids Res..

[19] Xiang X., You X.-M., Li L.-Q. (2018). Expression of HSP90AA1/HSPA8 in hepatocellular carcinoma patients with depression. OncoTargets Ther..

[20] Huang Y., Huang J., Huang Y., Gan L., Long L., Pu A., Xie R. (2020). TFRC promotes epithelial ovarian cancer cell proliferation and metastasis via up-regulation of AXIN2 expression. Am. J. Canc. Res..

[21] Milde-Langosch K. (2005). The Fos family of transcription factors and their role in tumourigenesis. Eur. J. Canc..

[22] Li D., Ilnytskyy Y., Kovalchuk A., Khachigian L.M., Bronson R.T., Wang B., Kovalchuk O. (2013). Crucial role for early growth response-1 in the transcriptional regulation of miR-20b in breast cancer. Oncotarget.

[23] Jia J.-J., Geng W.-S., Wang Z.-Q., Chen L., Zeng X.-S. (2019). The role of thioredoxin system in cancer: strategy for cancer therapy, Cancer Chemother. Pharmacol.

[24] Zhu Z., Song J., Guo Y., Huang Z., Chen X., Dang X., Huang Y., Wang Y., Ou W., Yang Y., Yu W., Liu C.-Y., Cui L. (2020). LAMB3 promotes tumour progression through the AKT-FOXO3/4 axis and is transcriptionally regulated by the BRD2/acetylated ELK4 complex in colorectal cancer. Oncogene.

[25] Baba Y., Iyama K.-I., Hirashima K., Nagai Y., Yoshida N., Hayashi N., Miyanari N., Baba H. (2008). Laminin-332 promotes the invasion of oesophageal squamous cell carcinoma via PI3K activation. Br. J. Canc..

[26] Duran A., Linares J.F., Galvez A.S., Wikenheiser K., Flores J.M., Diaz-Meco M.T., Moscat J. (2008). The signaling adaptor p62 is an important NF-kappaB mediator in tumorigenesis. Canc. Cell.

[27] Hsu L.-S., Huang R.-H., Lai H.-W., Hsu H.-T., Sung W.-W., Hsieh M.-J., Wu C.-Y., Lin Y.-M., Chen M.-K., Lo Y.-S., Chen C.-J. (2017). KLF6 inhibited oral cancer migration and invasion via downregulation of mesenchymal markers and inhibition of MMP-9 activities. Int. J. Med. Sci..

[28] Liang G., Wolfgang C.D., Chen B.P., Chen T.H., Hai T. (1996). ATF3 gene. Genomic organization, promoter, and regulation. J. Biol. Chem..

[29] Karst A.M., Gao K., Nelson C.C., Li G. (2009). Nuclear factor kappa B subunit p50 promotes melanoma angiogenesis by upregulating interleukin-6 expression. Int. J. Canc..

[30] Wang Z., Xu D., Ding H.-F., Kim J., Zhang J., Hai T., Yan C. (2015). Loss of ATF3 promotes Akt activation and prostate cancer development in a Pten knockout mouse model. Oncogene.

[31] Cairns J., Fridley B.L., Jenkins G.D., Zhuang Y., Yu J., Wang L. (2018). Differential roles of ERRFI1 in EGFR and AKT pathway regulation affect cancer proliferation. EMBO Rep..

[32] He P., Li K., Li S.-B., Hu T.-T., Guan M., Sun F.-Y., Liu W.-W. (2018). Upregulation of AKAP12 with HDAC3 depletion suppresses the progression and migration of colorectal cancer. Int. J. Oncol..

