# Supplementary material for: Analysis of differentially expressed genes responsible for the suppressive effect of anisomycin on cell proliferation of DLD-1 cells
Source: Biochem Biophys Rep. 2021 Jun 5;27:101038. doi: 10.1016/j.bbrep.2021.101038 (PMC8190440; doi:10.1016/j.bbrep.2021.101038)
Supplement: Multimedia component 1 [file mmc1.docx]

**Supplemental Table 1. The KEGG pathway analysis for the anisomycin-treated groups with different incubation time compared with the 24 h-treatment group**

|  | | Incubation time | Pathway | Adjusted *P* value |
| --- | --- | --- | --- | --- |
| Pathways affected by up-regulated genes | | 3 h | TNF signaling pathway | 1.40E-08 |
|  |  |  | Transcriptional misregulation in cancer | 1.40E-08 |
|  |  |  | Apoptosis | 2.77E-07 |
|  |  |  | NF-kappa B signaling pathway | 7.95E-07 |
|  |  |  | Micro RNAs in cancer | 8.95E-07 |
|  |  | 6 h | Hippo signaling pathway | 5.57E-07 |
|  |  |  | Endocytosis | 6.10E-07 |
|  |  |  | Apoptosis | 7.32E-07 |
|  |  |  | TNF signaling pathway | 7.32E-07 |
|  |  | 12 h | Small cell lung cancer | 6.37E-09 |
|  |  |  | Pathways in cancer | 5.01E-07 |
|  | | | | |
| Pathways affected by down-regulated genes | | 3 h | N/A | more than 1.00E-03 |
|  |  | 6 h | Metabolic pathway | 2.14E-08 |
|  |  |  | Lysosome | 4.26E-08 |
|  |  | 12 h | Metabolic pathway | 2.47E-26 |
|  |  |  | Protein processing in endoplasmic reticulum | 1.78E-20 |
|  |  |  | Lysosome | 1.95E-18 |
|  |  |  | N-glycan biosynthesis | 1.73E-08 |
